# Bioactive indanes: insight into the bioactivity of indane dimers related to the lead anti-inflammatory molecule PH46A

**DOI:** 10.1111/jphp.13269

**Published:** 2020-04-16

**Authors:** Kit Chan, Neil Frankish, Tao Zhang, Abdulilah Ece, Aoife Cannon, Jacintha O'Sullivan, Helen Sheridan

**Affiliations:** School of Pharmacy and Pharmaceutical Sciences and Trinity Biomedical Sciences Institute (TBSI), Trinity College Dublin, Dublin 2, Ireland; School of Food Science and Environmental Health, Technological University Dublin, Dublin 1, Ireland; Department of Pharmaceutical Chemistry, Faculty of Pharmacy, Biruni University, Topkapi-Istanbul, Turkey; Department of Surgery, School of Medicine, Trinity Translation Medicine Institute (TTMI), St James's Hospital, Dublin 8, Ireland

**Keywords:** colitis, cytokine profiling, inflammatory bowel disease, indanes, lipoxygenase, nitric oxide

## Abstract

**Objectives:**

PH46A (**1**) demonstrates significant anti-inflammatory activity in phenotypic models but its mechanism and site of action have been elusive. Current study focused on the bioactivity of PH46 (**2**) and related novel indane dimers (**6-10**) to investigate the impact of changes in substitution and stereochemistry at the C-1 and C-2 positions of the PH46 (**2**) scaffold.

**Methods:**

Cytotoxicity profiles of compounds were established using THP-1 macrophages and SW480 cells. Effects of the compounds were then evaluated at 10 µm using 5-lipoxygenase (LOX) and 15-LOX enzymes, and 5-LOX binding was evaluated *in silico* against NDGA, nitric oxide (NO) released from LPS-induced SW480 cells and cytokines in THP-1 macrophages (IL-6, IL-1β, TNF-α and IFN-γ) and in SW480 cells (IL-8).

**Key findings:**

PH46 (**2**) and **7** cause reduction in NO, inhibition of 5-LOX with high binding energy and no cytotoxicity effects in THP-1 macrophages and SW480 cell lines (up to 50 µm). The cytokine profiling of the series demonstrated inhibition of IL-6 and TNF-α in THP-1 macrophages together with IL-8 in SW480 cells.

**Conclusions:**

The observed profile of cytokine modulation (IL-6/ TNF-α, IL-8) and inhibition of release of NO and 5-LOX may contribute to the *in vivo* effects demonstrated by indane dimers and PH46A (**1**) in murine models of colitis.

## Introduction

**Figure 1:**
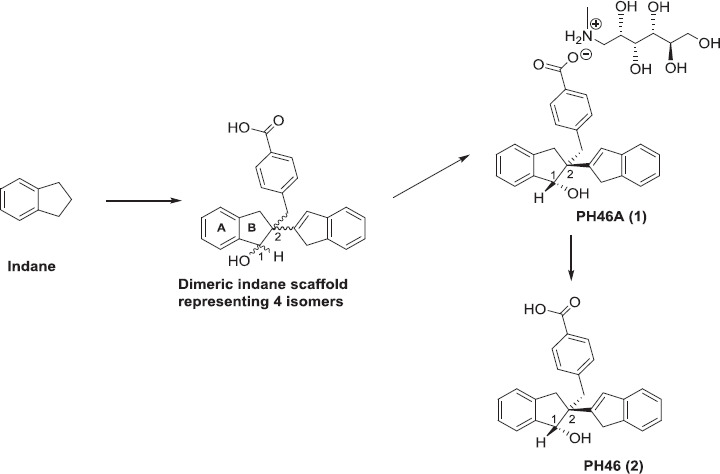
Indane, isomeric indane dimer scaffold; PH46A (1) and PH46 (2).

The indane (2,3-dihydro-1*H*-indene) ring system consists of a combination of aromatic benzene and aliphatic cyclopentane ring as shown in Figure 1. This gives a broad diversity of chemical entities with indane as the core structure. The ring system can accommodate substituents on its aliphatic and on its aromatic moiety which can modulate the biological properties of the molecule.^[1]^ This versatility has contributed to indane being identified as a privileged structure.^[2]^ The indane ‘scaffold’ also commonly occurs naturally or in medicinal chemistry as indanones, indandiones and indanols.^[3]^

The current study is based on molecules that have been developed in our group over the last 20 years. These studies centred on the synthesis and evaluation of the pharmacological activity of nature identical and synthetically modified indanes, indanones and dimeric indane. One of these molecules, PH46A (**1**) or 6-(Methylamino)hexane-1,2,3,4,5-pentanol 4-(((1S,2S)-1-hydroxy-2,3-dihydro-1H,1′H-[2,2-biinden]-2-yl)methyl)benzoate) has advanced to clinical trial (ISRCTN90725219) (singular). PH46A (**1**) is a first in class with a novel scaffold.^[4]^ In early studies, simple monomeric indanes, with a structure based on the natural pterosin scaffold, were designed and shown to demonstrate smooth muscle relaxant and mast cell stabilisation effects.^[5–8]^ Subsequent work led to the development of dimeric indane molecules which demonstrated mast cell stabilisation activities, with twice the order of magnitude than the monomeric indane series.^[9,10]^ At that point we considered the potential of the series for their clinical use as new chemical entities in the prevention of asthma based on their combined mast cell stabilisation and muscle relaxant properties.

As studies progressed, we revealed that the series of lead molecules also had significant anti-inflammatory and immune-modulating activity. On evaluation *in vivo*, the lead compounds showed significant activity in several models of murine colitis, for example, PH46 (**2**) reduced colon cytokine levels of TNFα, IL-6 and IL1b in DSS colitis mice.^[4]^ These molecules also prevented histological damage to colons. From these results, a lead molecule PH46A (**1**) demonstrated efficacy in two murine models of inflammatory bowel disease (IBD): an acute dextran sulphate sodium (DSS) model of murine colitis and the more chronic spontaneous model of *IL-10^−/−^* colitis mice.^[4]^ The scaffold of PH46A (**1**) incorporates the indane skeleton and is the *N*-glucamine salt of a single enantiomer PH46 (**2**) of this novel drug with *1S, 2S* stereochemistry at the C-1 and C-2 positions. The success of PH46A (**1**) in ameliorating the effects of *in vivo* activity in the DSS and *IL-10^−/−^* murine model of colitis has led to its potential as a therapeutic agent in IBD. It is interesting to note that of the four possible enantiomers forming the isomeric dimeric indane scaffold that with the *1S, 2S* stereochemistry at C-1 and C-2 (as shown in Figure 1) gave optimal results in the *in vivo* colitis studies.

Despite the impressive *in vivo* results observed for PH46A (**1**), the absence of understanding the molecular target has prompted further investigation into the activity of PH46A (**1**) and PH46 (**2**), and some analogues in order to build understanding of what may be a novel, complex mechanism of action or biological fingerprint. Within the series there is currently no clear understanding of the effect of substitution at the C-1 and C-2 positions on the pharmacological activity of analogous compounds, although our earlier work has identified what appears to be an optimal stereochemical configuration for the enantiomers of PH46 (**2**).

In the current study, for the first time, we have characterised the bioactivity of these novel indane dimers in *in vitro*, *in vivo* and *in silico* models. This allows us to understand the relationship of stereochemistry and substitution at the C-1 and C-2 positions on indanol ring B on biological activity (Figure 1). The results of the bioactivities have enabled us to progress to a deeper understanding in the pharmacological fingerprint of the indane dimers in the development of potential therapeutic agents in IBD and other related inflammatory diseases.

## Materials and Methods

### Synthesis of indane dimers

PH46A (**1**) and PH46 (**2**) were synthesised according to the experimental method previously reported.^[4]^ The key compounds of interest **6** and **7** were synthesised from **3,** with intermediate compounds** 4** and **5**^[11,12]^ (Scheme S1). Compound **8** was supplied by Trino Therapeutics Ltd and was used as intermediate compound to synthesise compounds **9** and **10** (Scheme S2).^[9,10,13]^

### Measurement of cytotoxicity of test molecules on THP-1 and SW480 cells using acid phosphatase assay

THP-1 monocytes (ATCC TIB-202; LGC Standards, Middlesex, UK) and SW480 cells were seeded in 96-well plates at concentration 4 × 10^4^ per well in 100 µl. For THP-1 monocytes, the cells were differentiated with 200 nm of PMA (Sigma-Adrich, Darmstadt, Germany) for 48 h. The PMA-differentiated macrophages were cultured and left at resting period for 3 days (PMAr stage).^[14]^ For SW480, the cells were seeded in 96-well plates 24 h prior to drug incubation at 10 µm. After 24 h, the media were removed and each well was washed with 100 µl of PBS and the step was repeated twice. After washing, 100 µl of freshly prepared phosphatase substrate (10 mm  *p*-nitrophenyl phosphate in 0.1 m sodium acetate, 0.1% Triton X-100, pH 5.5) was added into each well. The plates were incubated in the dark at 37°C and 5% CO_2_ for 1 h. The reaction was stopped by adding 20 µl of Na OH. Readings of the wells were recorded at 405 nm.^[15]^

### Measurement of 5-LOX inhibition by test molecules using UV-Vis spectrophotometric assay

Briefly, 100 µl of 2.75-unit human recombinant 5-LOX (final concentration is 0.5 unit/ml) (Cayman Chemicals, Ann Arbor, MI, USA) was pre-incubated with 50 µl of compounds/standard inhibitor/buffer, 100 µl of 2.2 mm CaCl_2_ (final concentration is 0.4 mm), 100 µl of 132 µg/ml L-α-phosphatidylcholine type II-S (final concentration is 24 µg/ml) and 100 µl of 1.1 mm ATP (final concentration is 0.2 mm) at RT for 5 min in the dark. The reactions were started by adding 100 µl of 275 µm arachidonic acid (final concentration is 50 µm) to the mixture in quartz microcuvettes. The mixture was gently mixed by pipetting three times and the increase in absorbance at 234 nm was measured using the Shimadzu UV-1602 spectrophotometer. The compounds were screened using one concentration point to determine the percentage inhibition by following the formation of the conjugated diene product 5-HpETE at 234 nm (ε = 25 000 M^−1^ cm^−1^).^[16]^

### Measurement of 15-LOX inhibition by test molecules using FOX enzymatic assay

In the sample wells, 50 µl of rabbit reticulocyte 15-LOX-1 (2 µl from purified stock at 5000 units/ml; Enzo Life Sciences, Farmingdale, NY, USA) was pre-incubated with 50 µl of compounds/standard inhibitors (final concentration is 30 µm) at 25°C for 5 min. The control well consisted of 50 µl of rabbit reticulocyte 15-LOX-1 and 50 µl of buffer in 0.2% DMSO. The reaction was initiated by adding 50 µl of arachidonic acid (final concentration of 50 µm) into both the sample and control wells. The reaction mixture was incubated at 25°C for 15 min in the dark. The reaction was terminated by adding 100 µl of FOX reagent after reaction period of 15 min. The FOX reagent contained 30 mm sulphuric acid, 100 µm xylenol orange and 100 µm ferrous (II) sulphate in MeOH : water (9 : 1). The absorbance readings were measured at 584 nm after 30 min of colour development at RT.^[17]^

### Measurement of nitric oxide inhibition by test molecules using GREISS assay

SW480 cells were stimulated with 1 µg/ml of *Salmonella enterica* serotype *typhimurium* LPS and IFN-γ^[18]^ (Sigma-Aldrich). The treatment of cells with LPS was carried out for 48 h, at 37°C under 5% CO_2_. After treatment, the culture supernatant was collected and immediately tested for nitrite quantitation using GREISS reagent. Equal volume of fresh culture supernatant and sulphanilamide solution was loaded into each well of 96-well plates. The mixture was incubated for 10 min, in the dark at RT. Next, same volume of *N*-1-napthylethylenediamine dihydrochloride (NED) was added into each well and incubated for 20 min. A purple/magenta colour was formed in the wells and the absorbance was recorded at 540 nm. A standard curve (0–100 µm) of NO_2_^−^ was developed using known standards as provided by the kit.

### Cytokine profiling using THP-1 and SW480 cells

#### THP-1 macrophages

THP-1 monocytes were seeded in 24-well plates at density of 2.5 × 10^5^ cells per well in 500 µl. The differentiation process and resting period of the THP-1 macrophages were carried out and adapted.^[14]^ After 1 h of drug exposure, the cells were stimulated with 1 µg/ml of *Escherichia coli* 0111: B4 LPS (Sigma-Aldrich). At 24 h, the media were collected from each well, transferred into 2.0-ml microcentrifuge tubes and centrifuged at 17 950 *g* for 10 min at 4°C. Supernatant collected was analysed using MSD V-PLEX Proinflammatory Panel 1 Human (4-Plex) Cat. No K15052D and V-PLEX individual assay Cat. No. K151QUD kits (Meso Scale Diagnostics, Rockville, MD, USA).

#### SW480 cells

SW480 cells were seeded into 96-well plates at density 4 × 10^4^ cells/well and left to grow until 80% confluency. The cells were stimulated with 1 µg/ml of *E. coli* 0111: B4 LPS after 1 h of drug treatment. Supernatant was collected from each well at 24 h. The supernatant was transferred into 2.0-ml microcentrifuge tubes and centrifuged at 13 000 rpm for 10 min at 4°C. Supernatant collected was analysed using MSD U-PLEX Biomarker Group 1 Human (10-Plex) Cat. No. K15067L-2 (Meso Scale Diagnostics) plates.

### Molecular docking of test molecules using 5-LOX

As a first step in molecular modelling compounds **2**, **6**, **7**, **8**, **9** and **10** and the reference standard nordihydroguaiaretic acid (NDGA) were prepared using LigPrep^[19]^ tool of the Schrödinger^[19]^ software to generate the 3D structures. During the process, input ionisation states were retained and chiralities were determined from 3D structures. Final structures were minimised using optimised potential liquid simulations (OPLS3e) force field.^[20]^ Structure of the target protein also needs to be prepared before going further into *in silico* calculations. Accordingly, crystal structure of human 5-LOX (PDB ID: 3V99, 2.252 Å) in complexed with arachidonic acid was retrieved from RSCB protein data bank and subjected to Protein Preparation Wizard module of Schrödinger's.^[21,22]^ Bond orders were assigned, missing hydrogens were added, chain B, all water molecules and heteroatoms except arachidonic acid were removed. In the next step, the structure was optimised and further minimised using OPLS3e force field to avoid steric clashes. Arachidonic acid was taken as a reference to define the binding site of 5-LOX. A grid representing the binding site was generated using default settings. Glide XP (extra precision)^[23]^ module was used to dock the compounds into the binding pocket.

### Statistical evaluation

Statistical comparisons (GraphPad Prism 5.01; GraphPad Software, San Diego, CA, USA) were made using one-way ANOVA with *P* < 0.05 considered as statistically significant. Dunnett's Multiple Comparison Test was chosen to compare between the control group and samples groups. Subsequently, Bonferroni's Multiple Comparison Test was used to compare the samples groups in the cytokines ‘profiling to evaluate the SARs’. The results were expressed as the mean ± standard error mean (*n* = 3 experiments performed on different days), unless otherwise stated. Asterisks indicate the value is significant (*P < 0.05; **P < 0.01; ***P < 0.001).

## Results

### Cytotoxicity effects on THP-1 and SW480 cells

**Figure 2:**
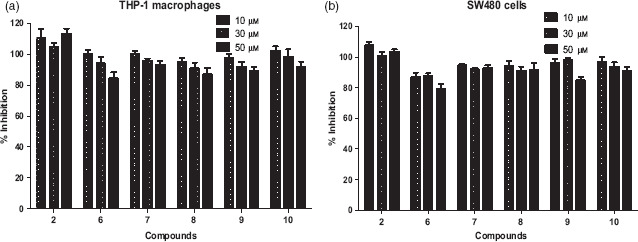
Preliminary cytotoxicity screening at 10, 30 and 50 μm determined by acid phosphatase assay after incubation for 24 h: (a) THP-1 macrophages and (b) SW480 cells. Results were expressed as mean ± standard error mean (SEM; *n* = 4). No significant (*P < 0.05) values were observed.

In the current study, 30 µm was selected as the tested concentration with addition of one higher and one lower concentrations: 50 and 10 µm, respectively. From Figure 2, it was observed that none of the compounds exhibit any significant cytotoxic activities at the highest concentration of 50 µm in either THP-1 macrophages or SW480. This study allowed us to establish a dose range for incubation of test compounds in a non-toxic dose range.

### Measurement of 5-LOX and 15-LOX inhibition

**Figure 3:**
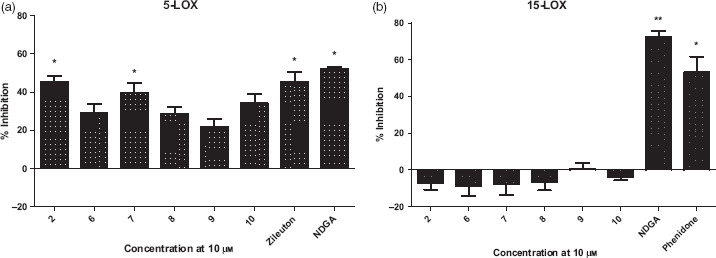
LOXs inhibitory activities of indane dimers and standard inhibitors using; (a) 5-LOX (b) 15-LOX. Results were expressed as mean ± standard error mean (SEM; *n* = 3). Asterisks indicate the value is significant (*P < 0.05; **P < 0.01). LOX, lipoxygenase.

Of the compounds screened at 10 µm, only PH46 (**2**) and methylene analogue **7** inhibited 5-LOX significantly at 45.30 ± 5.28% and 39.71 ± 4.95%, respectively, *P* < 0.05. Results were comparable to the degree of inhibition of zileuton at 45.22 ± 5.36%, *P* < 0.05. The % inhibition of control NDGA was observed at 51.92 ± 1.01%, *P* < 0.05, as shown in Figure 3. The indane dimers did not give any significant inhibition of 15-LOX (Figure 3) and control NDGA at 10 µm inhibited 15-LOX activity at 72.43 ± 3.25%. The Z′-factor in this 96-well microplate ferrous oxidation-xylenol orange (FOX) assay was determined as 0.891 which demonstrated the suitability for mid-throughput to high-throughput screening.^[24]^ The IC_50_ value for NDGA was determined at 4.61 ± 0.93 µm, similar to the literature values.^[25]^.

### Docking analysis using 5-LOX

As shown from Table 1, the active compounds against 5-LOX **2** and **7**, together with the reference compound NDGA, had the highest score. Besides a number of hydrophobic interactions, NDGA makes strong hydrogen bonds with Val671, HIS367, THR 364 and GLN363 in the binding pocket. In addition, a favourable T-shaped π- π interaction with HIS367 is also observed. Docking analysis of the active compound **2** indicates that the hydroxy group at C-1 position participates a hydrogen bonding with HIE550 of 5-LOX. No other hydrogen bond is observed but the carboxy group orients itself towards polar LYS409 suggesting a possible favourable interaction.

**Table 1:** Docking scores of nordihydroguaiaretic acid (NDGA) and compounds **2**, **6-10**

Compound	Docking Score (kcal/mol)
NDGA	−9.36
7	−6.30
2	−6.07
8	−5.63
6	−5.60
10	−5.21
9	−4.40

**Figure 4:**
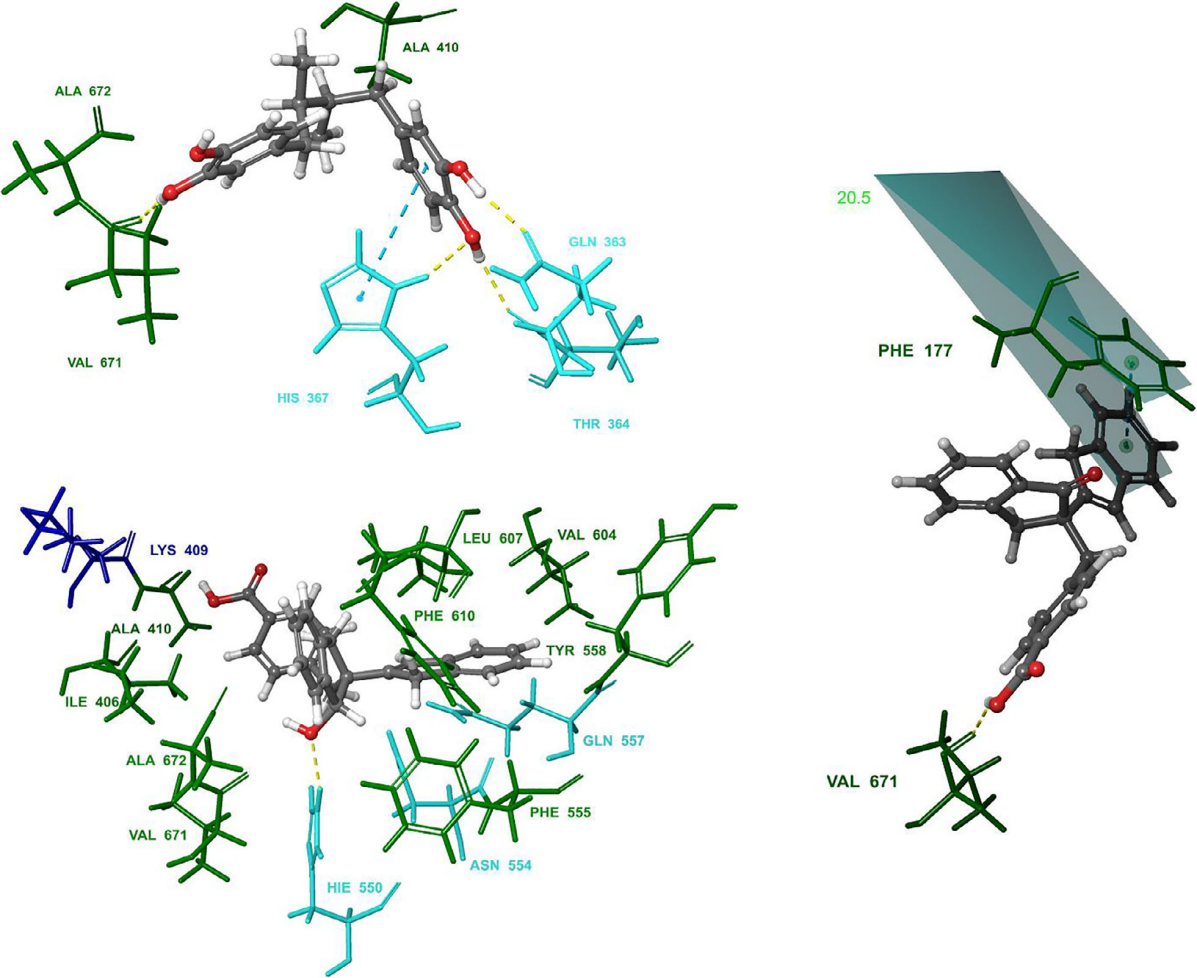
Calculated 3D binding interactions of nordihydroguaiaretic acid (NDGA) (top left), 2 (bottom left) and 6 (right) with 5-LOX. Amino acid residues are coloured by properties (Hydrogen bonds: yellow dashed lines; π-π interactions: cyan dashed lines; hydrophobic: green; polar: cyan; positively charged: purple). For the sake of clarity, only discussed crucial amino acids are shown. LOX, lipoxygenase.

Hydrophobic interactions of indene moiety with PHE610, LEU607, AL604 and THY558 also contribute to the binding of **2** and reduce the energy of protein–ligand complex which in turn enhances activity. In compound **6** where hydroxy group at C-1 position of **2** is replaced by carbonyl group, a new hydrogen bond is observed between carboxy group and VAL671 which may compensate the absence of favourable interaction of -OH group. However, compound **6** was observed to adopt a different orientation in the 5-LOX binding pocket. Most noticeable, an unfavourable face-to-face π-π interaction between indene ring and PHE177 is observed (the angle between the ring planes is 20.5°). Hence, the favourable hydrophobic interaction of indene moiety is now replaced with unfavourable π-π stacking interaction (Figure 4).

### Measurement of nitric oxide inhibition

**Figure 5:**
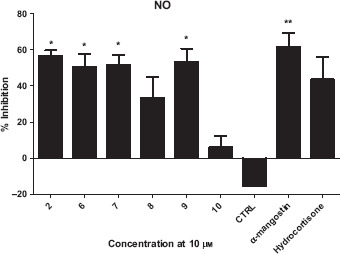
Effects of indane dimers at 10 μm on nitric oxide (NO) released from SW480 cells. Results were expressed as mean ± standard error mean (SEM; *n* = 3). Asterisks indicate the value is significant (*P < 0.05; **P < 0.01).

Preliminary screening of the indane dimers (Figure 5) showed comparable inhibition to the positive control, hydrocortisone at 10 µm (43.55 ± 12.27%) but did not show statistical significance due to a slight increase in SEM at *n* = 3. A second positive control, the natural product and nitric oxide (NO) inhibitor, α-mangostin^[26]^ gave an inhibition of 61.63 ± 7.50% with *P* < 0.01. Of the analogues examined, PH46 (**2**) exhibited the highest inhibition with 56.50 ± 3.09%. *Para*-carboxybenzyl derivatives **6, 7** and the *para*-unsubstituted benzyl **9** also inhibited NO production. IC_50_ values were determined for analogues which inhibited at greater than 50% at the selected 10 µm concentration. The IC_50_ value of α-mangostin was determined at 8.87 ± 1.18 µm and this value is close to the reported values of IC_50_: 12.2 µm.^[26]^ The IC_50_ value of hydrocortisone was 48.66 µm consistent with literature values.^[27]^ PH46 (**2**) displayed the lowest IC_50_ value of 19.70 ± 7.88 µm of all the analogues, followed by compound **9** (IC_50_: 20.49 ± 2.89 µm).

### Cytokine profiling using THP-1 and SW480 cells

**Figure 6:**
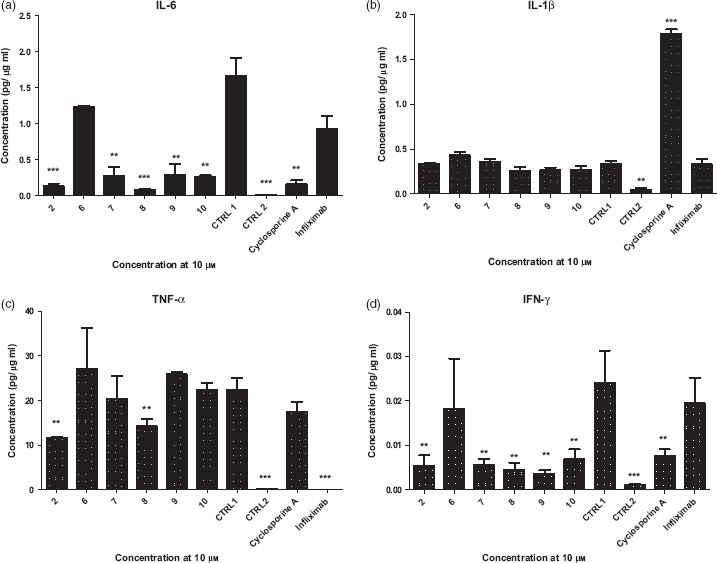
Cytokine profiling from THP-1 macrophages of indane dimers at 10 μm; (a) IL-6 (b) IL-1β (c) TNF-α (d) IFN-γ. Results were expressed as mean ± standard error mean (SEM; *n* = 3). Asterisks indicate the value is significant (*P < 0.05; **P < 0.01; ***P < 0.001).

IL-6 plays a central role in the maintenance of chronic intestinal inflammation in IBD.^[28]^ The level of IL-6 in THP-1 macrophages stimulated with LPS without drug treatment was 1.666 ± 0.256 pg/µg ml in CTRL 1 as shown in Figure 6. CTRL 2 represented the level of IL-6 in cells without LPS stimulation, at 0.0015 ± 0.016 pg/µg ml. The Cyclosporine A control significantly reduced the production of IL-6 from the value of 1.666 ± 0.256 pg/µg ml to 0.161 ± 0.053 pg/µg ml, with *P* < 0.001. Infliximab did not inhibit IL-6 significantly from the value of 1.666 ± 0.256 pg/µg ml to 0.924 ± 0.180 pg/µg ml. Infliximab was used as the positive control as it inhibits the production of TNF-α and is used as anti-TNF-α therapy for IBD treatment clinically.^[29,30]^ PH46 (**2**) and compound **8**, its unsubstituted benzyl analogue, displayed potent IL-6 inhibition with levels of inhibition of 92.07% (0.132 ± 0.038 pg/µg ml) and 95.49% (0.075 ± 0.019 pg/µg ml), respectively, over CTRL 1.

Cytokine IL-1β triggers intestinal inflammation by augmenting the recruitment of granulocytes and the activation of intestinal epithelial cells (IECs).^[31]^ In Figure 6, the level of IL-1β in CTRL 1 was 0.329 ± 0.042 pg/µg ml vs level of IL-1β in CTRL 2 at 0.045 ± 0.015 pg/µg ml. Ciclosporin A stimulated production of IL-1β significantly with *P* < 0.001, at 1.784 ± 0.052 pg/µg ml. Infliximab did not have significant inhibitory effect on IL-1β, with production level at 0.329 ± 0.059 pg/µg ml. No significant inhibition was observed by analogues in the study. TNF-α has been shown to induce various inflammatory mediators in the inflamed mucosa in IBD.^[32]^ In the cytokine profiling for TNF-α, the level of TNF-α for CTRL 1 was measured at 22.305 ± 2.753 pg/µg ml, whereas CTRL 2 (without LPS stimulation) measured at 0.060 ± 0.019 pg/µg ml as shown in Figure 6. Infliximab at 10 µm effectively inhibited the production of TNF-α with % inhibition of 99.98%, measuring at 0.002 ± 0 pg/µg ml, p < 0.001. Cyclosporine A slightly inhibited the release of TNF-α at 17.57% (17.460 ± 2.249 pg/µg ml) at 10 µm, but the value was not statistically significant. The profiling of TNF-α was like IL-6 where PH46 (**2**) and **8** inhibited TNF-α at 45.18% (11.613 ± 0.162 pg/µg ml) and 32.99% (14.193 ± 1.685 pg/µg ml) respectively. These compounds both have a hydroxyl group with the *S*-configuration at C-1 position which may play a role in assisting TNF-α inhibitory activity.

Studies have shown that the production of IFN-γ is increased in lamina propria and T cells in patients with IBD.^[33]^ In this study, the release of IFN-γ was low compared with the other three analytes under the same experimental conditions. The level of IFN-γ in LPS-stimulated THP-1 macrophages was 0.024 ± 0.007 pg/µg ml and 0.001 ± 0.000 pg/µg ml in THP-1 macrophages without LPS stimulated. Cyclosporine A inhibited IFN-γ with % inhibition of 68.05% (0.008 ± 0.001 pg/µg ml, *P* < 0.01) as shown in Figure 6. It was observed that the indane dimers inhibited production of IFN-γ significantly, with activity ranging from 70.0 to 85.0% in all the molecules except **6** and the activities are comparable with cyclosporine A. Some groups exhibited greater variability due to the low detection of IFN-γ.

**Figure 7:**
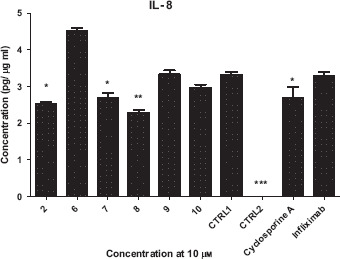
Effects of indane dimers at 10 μm on IL-8 from LPS-induced SW480 cells. Results were expressed as mean ± standard error mean (SEM; *n* = 3). Asterisks indicate the value is significant (*P < 0.05; **P < 0.01; ***P < 0.001).

IL-8 is a potent angiogenic factor in colon wound healing, colitis and cancer. In patients with IBD, IL-8 is overexpressed and generally localised to areas of active inflammation and tissue damage.^[34,35]^ Overall the inhibition over CTRL 1 values range from 12.70 to 25.0% for the mentioned molecules as shown in Figure 7. CTRL 1 (with LPS stimulation) was measured at 3.324 ± 0.076 pg/µg ml vs CTRL 2 (without LPS stimulation) at 0 pg/µg ml, *P* < 0.001. Cyclosporine A inhibited IL-8 with *P* < 0.05, measuring at 2.689 ± 0.293 pg/µg ml. The inhibitory effects observed with PH46 (**2**) and compound **8** on IL-6 and TNF-α release from THP-1 macrophages were also observed on the release of IL-8 from SW480 cells with *P* < 0.05, 23.92% (2.529 ± 0.058 pg/µg ml) and 31.17% (2.288 ± 0.059 pg/µg ml) respectively. In addition, compound **7** inhibited IL-8 measuring at 19.49% (2.676 ± 0.142 pg/µg ml), *P* < 0.05. The effect of this molecule on IL-6 release from THP-1 macrophages was less potent but statistically significant.

## Discussion

The effects of PH46 (**2**) and the indane dimers (**6-10**) on 5-LOX, NO and on the production of inflammatory mediators from THP-1 macrophages and SW480 cells were investigated. PH46 (**2**) was selected for these studies, to facilitate comparison with other close analogues for which there was no salt available or for which suitable salts could not be prepared. The selection of PH46 (**2**) over PH46A (**1**) is justified given that *in vitro* and clinical data has shown that PH46A (**1**) dissociates in solution to PH46 (**2**) and the *N*-glucamine salt.^[4]^

To establish the concentration range of the indane dimers that could be used in *in vitro* cell studies compounds, cell viability screening of the novel compounds was evaluated at 10, 30 and 50 µm against the THP-1 human monocytic cell line, which is a model for the evaluation of immune-modulating effects of compounds^[36]^ and the SW480 cells.^[37]^ The optimised concentration in high-throughput drug screening is 30 µm for cell-based assays to minimise the effects of false-positive results.^[38]^ None of the compounds exhibits any significant cytotoxic activities at the highest concentration of 50 µm in either THP-1 macrophages or SW480.

Earlier studies have shown that the *in vivo* anti-inflammatory effects of the indane dimers (**1**, **2** and **8**) are independent of cyclooxygenase (COX) (Frankish & Sheridan unpublished results). The zileuton (**12**), is a bioisostere of indane, and is also an orally active inhibitor of the 5-LOX enzyme and was used in this study as a positive control. The ability of bioisosteres to elicit similar biological activity has been attributed to common physicochemical properties.^[39]^ The 5-LOX plays a unique role in the production of leukotrienes which makes it a potential therapeutic target for inflammatory conditions like asthma, rheumatoid arthritis, psoriasis and IBD.^[40]^ Due to the involvement of 5-LOX in IBD and the profound activity observed for PH 46A (**1**) and PH 46 (**2**) and its analogues in *in vivo* colitis models,^[4]^ the activity of PH46 (**2**), the indane dimers (**6-10**) and positive controls **12** and phenidone was evaluated on 5-LOX and 15-LOX enzymes, to see if LOX activity is part of the pharmacological fingerprint of the series. Furthermore, molecular docking calculations were performed to further support biological activity results towards 5-LOX. Docking calculations revealed that binding site of 5-LOX is composed of both hydrophilic and hydrophobic amino acid residues.

Active inflammation of the colon lining during IBD is associated with increased production of cytokines released by the colonic cells, macrophages and neutrophils. These cytokines such as IFN-γ and TNF-α induce the release of inducible NO synthase (iNOS) via nuclear factor-κB (NF-κB) pathway which results in increased production of NO.^[41]^ NO overproduction via iNOS upregulation by intestinal epithelium has been consistently associated with IBD.^[42]^ The effects of compounds, PH46 (**2**) and the indane dimers (**6-10**) on NO inhibition were evaluated in this study, to elucidate if such activity may be a contributing factor to the observed beneficial *in vivo* effects of the novel scaffold in murine colitis models. Although this study does not probe the mechanism of NO inhibition, and will need to be expanded, it suggests that NO inhibition may be a significant activity for the lead molecule PH46 (**2**) and may be a contributing factor to the effects observed in *in vivo* models.

To understand the impact of the indane dimer scaffold on cytokine production, we evaluated the effects of PH46 (**2**) and the related dimers (**6-10**) on the four primary cytokines released by the THP-1 macrophage cell line; TNF-α, IL-1β, IL-6 and IFN-γ, and on IL-8 released from SW480 cells. The indanes screened initially at 10 µm with drug incubation for 24 h. The total protein for the samples was determined. Measurement of each cytokine was represented by the concentration of each cytokine (pg/ml), expressed in relative to total protein quantified (µg) using BCA assay to give the final unit in pg/µg ml. Infliximab and cyclosporine A were used as positive controls similar to the previous study^[43]^ and they are known as standard medications used to treat IBD. The results obtained for the quantification of IL-8 in SW480 showed that there was a statistically significant inhibition of IL-8 release by some of the indane dimers with a methylene group at C-2 position.

The current study focused on probing activities that contribute to the pharmacological fingerprint of PH46 (**2**) and related molecules that might explain activity observed in *in vivo* phenotypic models. The study of the analogous series also aimed at understanding how substitution and stereochemistry at the C-1 and C-2 position of the B ring in the indane dimer scaffold might contribute to the observed biological activity. Specifically, we investigated the impact of subtle modification at C-1 and C-2 positions of the B ring in the dimers. The indane dimers, PH46 (**2**) and its C-1 methylene derivative **7** inhibited 5-LOX significantly (p < 0.05). These compounds have the *S*-*para*-carboxybenzyl substituent at C-2. The molecules differ in substitution at C-1, where **7** has no OH group, but both molecules retain sp3 hybridisation at this position, which may impact on binding with the isozymes. The observed inhibition of 5-LOX was further supported during molecular docking analysis which has shown highest docking scores for indane dimers PH46 (**2**) and **7**.

None of the test compounds showed a binding affinity with 15-LOX (Table 2). The cytokine profiling using THP-1 macrophages (IL-6, IL-1β, TNF-α and IFN-γ) and SW480 cells (IL-8) has identified a few key compounds with optimal substituents at the C-1 and C-2 positions. The two most active indanes **2** and **8** with the hydroxyl group at C-1 had been shown to have potential for the treatment of IBD in previous studies.^[4,9]^ This study shows that they inhibit the key mediators IL-6 and TNF-α in THP-1 macrophages and IL-8 in SW480 cells.

**Table 2:** Effects on novel indane dimers and standard inhibitors with significant values (p < 0.05) on the target IBD-related factors

	LOXs		THP-1 macrophages					SW480 cells		
	5-LOX	15-LOX	Cytotoxicity	IL-6	IL-1β	TNF-α	IFN-γ	Cytotoxicity	IL-8	NO
2	**Y**			**Y**		**Y**	**Y**		**Y**	**Y**
6										**Y**
7	**Y**			**Y**			**Y**		**Y**	**Y**
8				**Y**		**Y**	**Y**		**Y**	
9				**Y**			**Y**			**Y**
10				**Y**			**Y**			
Nordihydroguaiaretic acid (NDGA)	**Y**	**Y**								
Cyclosporine A				**Y**	**P**		**Y**		**Y**	
Infliximab						**Y**				

**Y**-inhibits; **P**-potentiates.

LOX, lipoxygenase.

The activity of **2**, **6** and **7** with the *para*-carboxybenzyl substituent at the C-2 position, and the activity of **9**, with just the benzyl group at C-2 suggest that the *para*-carboxy group does not contribute significantly to NO inhibition. The substituent at C-1 conferring the greatest activity for indane dimers is an *S*-hydroxyl group as observed in **2**, the most active compound. Overall, the effects of PH46 (**2**) and the novel analogues (**6**-**10**) on the key mediators with significant values at p < 0.05 are summarised in Table 2.

## Conclusion

The biological fingerprint of PH46 (**2**) and the related novel indane dimers (**6**-**10**) has been evaluated in *in vitro*, *in vivo* and *in silico* models and these studies have contributed to our understanding of this novel class of molecule. The cytokine profiling of the series demonstrated inhibition of IL-6 and TNF-α in THP-1 macrophages and IL-8 in SW480 cells. PH46 (**2**) and **7** cause a statistically significant reduction in NO production and reduction in 5-LOX activity in the arachidonic acid pathway. These activities should be considered to form part of the indane dimer biological fingerprint and may contribute to the *in vivo* effects demonstrated by PH46 (**2**) in murine models of colitis. PH46 (**2**) reduced DAI in the acute 5% DSS and IL-10 -/- murine model of colitis and significantly reduced MPO activity, a measure of inflammatory cell infiltration and inflammatory colon cytokines (IL-6, IL-1β and TNF-α).^[4]^ The observation of inhibition of NO and 5-LOX by PH46 (**2**) and other related indane molecules warrants further study and may help to elucidate a clearer understanding of the relationship between the reduction in inflammation in the *in vivo* models and the decreased level of cytokines observed. Further investigations will lead to a deeper understanding of the pharmacological fingerprint to identify potential development of anti-inflammatory therapeutic agents.

## Declarations

### Conflict of interest

The Author(s) declare(s) that they have no conflicts of interest to disclose.

### Author contributions

KC and TZ synthesised and characterised the compounds discussed in this manuscript. KC performed all biological assays with assistance from AC on the multiplex assays. AE performed the 5-LOX molecular docking analysis. HS, NF and JOS were involved in all aspects of the project design, supervision of chemical and biological experiments and data interpretation. SW-480 cell line was a gift from JOS. The manuscript was written through contributions of all authors. All authors have given approval to the final version of the manuscript.

## Supplementary Material

jphp13269-sup-0001-AppendixS1 **Appendix S1.** Materials, chemicals and synthesis of indane dimers.
