# Supplementary material for: Bioactive indanes: insight into the bioactivity of indane dimers related to the lead anti-inflammatory molecule PH46A
Source: J Pharm Pharmacol. 2020 Apr 16;72(7):927–37. doi: 10.1111/jphp.13269 (PMC7497186; doi:10.1111/jphp.13269)
Supplement: jphp13269-sup-0001-AppendixS1 — Appendix S1. Materials, chemicals and synthesis of indane dimers. [file jphp13269-sup-0001-appendixs1.docx]

**Supporting Information**

**Materials**

Human recombinant 5-lipoxygenase (5-LOX), zileuton and nordihydroguaiaretic acid (NDGA) were obtained from Cayman Chemicals Co. (Ann Arbor, USA). Rabbit reticulocyte 15-lipoxyeganse-1 (15-LOX-1) was purchased from Enzo Life Sciences (New York, USA). The THP-1 (ATCC® TIB-202™) cell line was purchased from ATCC company via LGC Standards (Middlesex, UK). The SW480 cell line was a gift from Prof. Jacintha O’Sullivan. Xylenol orange (XO), indomethacin, phenidone, ferrous (II) sulfate, calcium chloride, sodium acetate, calcium ionophore A 23187, tween-20, Triton™ X-100, vanillin, 1-indanone, tris(hydroxymethyl) aminomethane, L-α-phosphatidylcholine (type II-S), adenosine 5′-triphosphate disodium salt (ATP), porcine arachidonic acid, bovine serum albumin (BSA), lipopolysaccharides (LPS) from *Escherichia coli* 0111:B4 and *Salmonella enterica* serotype *typhimurium*, human IFN-γ, phorbol 12-myristate 13-acetate (PMA), RIPA buffer, RPMI-1640 media with L-glutamine and sodium bicarbonate, penicillin-streptomycin, trypsin-EDTA, phosphate buffered saline (PBS, 0.10 mol L^−1^, pH 7.4), dimethyl sulfoxide (DMSO) were purchased from Sigma-Aldrich (Darmstadt, Germany). Pierce^TM^ BCA Protein Assay Kit, trypan blue solution, Gibco fetal bovine serum (FBS) SKU 10270106 were purchased from Thermo Scientific (Rockford, USA). *p*-nitrophenyl phosphate salt was purchased from VWR International (Dublin, Ireland). Quartz cuvettes for high UV transmission were purchased from Hellma Analytics (Müllheim, Germany). Meso Scale Discovery (MSD) V-PLEX™ Proinflammatory Panel 1 Human (4-Plex) (for IFN-γ, IL-1β, IL-6, TNF-α), MSD U-PLEX™ Biomarker Group 1 Human (10-Plex) (for IL-1β, IL-6, IL-8, IL-10, IL-17A, IL-17F, IL-22, IL-23, TNF-α, VEGF-A) were purchased from Meso Scale Diagnostics (Maryland, USA). Griess Reagent System kit was purchased from Promega Corporation (Madison, USA).

**Chemicals**

Chemical reagents and solvents were obtained from commercial suppliers and used without further purification. Starting compounds were obtained internally within the research group and compound identity and purity were tested before use. Proton Nuclear Magnetic Resonance (NMR) spectra were recorded at 27°C on Bruker DPX 400 MHz spectrometer, using solvents DMSO-d_6_ or CDCl_3_ and referenced relative to residual DMSO-d_6_ (δ = 2.50 ppm) or CHCl_3_ (δ = 7.26 ppm). Chemical shifts are reported in ppm and coupling constants (*J*) in Hertz. Carbon NMR spectra were recorded on the same instruments (100 MHz) with total proton decoupling. NMR spectra were analysed with Brucker TopSpin 3.5 NMR software. ESI mass spectra were acquired using a Waters Micromass LCT- time of flight mass spectrometer, interfaced to a Waters 2690 HPLC. The instrument was operated in positive or negative mode as required. EI mass spectra were acquired using a GCT Premier Micromass time of flight mass spectrometer. The instrument was operated in positive mode. Chemical Ionisation mass spectra were determined using a GCT Premier Micromass mass spectrometer in CI mode utilising methane as the ionisation gas. Flash chromatography was carried out using silica gel 230-400 mesh. TLC analysis was performed on pre-coated 60F_254_ slides and visualized by UV irradiation. Specific optical rotation was measured at 22°C in MeOH or CHCl_3_. Achiral HPLC analyses were performed on a Waters 600 system, equipped with an autosampler, binary pump (post-pump mixing), and PDA detector, and using Waters Nova-Pak C18 column (4 µm, 150 x 3.9 mm). The flow rate was 1 mL/min with isocratic elution with a mobile phase MeOH:water:TFA (80:20:0.1, *v/v/v*). For compounds **10** and **7**, The flow rate was 0.8 mL/min with isocratic elution with a mobile phase MeOH:water:TFA (90:10:0.1, *v/v/v*). Solvents (MeOH and water) and additives (TFA) were all of HPLC solvent grade. Detection was 254 nm and column temperature was at room temperature (RT). Samples were prepared at 0.5 mg/mL in the eluent mixture. 5 μL of samples were injected by autosampler for the analyses and detector wavelength of 254 nm was used.

The analyses were run at RT and the retention times (r. t.) given may differ slightly depending on temperature and concentration of the sample but the elution order of the compounds will remain unchanged. The details of the mobile phase and the r. t. are given below under each compound’s characterisation.

**Synthesis of indane dimers**

PH46A (**1**) and PH46 (**2**) were synthesised according to the experimental method previously reported ^4^. The key compounds of interest in the study **6** and **7** were synthesised from **3** as shown in Scheme S1. Methyl ester **4** was obtained via esterification using trimethylsilyldiazomethane in a mixture of toluene and MeOH from compound **3**. Subsequently, without purification, the alcoholic ester **4** was oxidised to ketone **5** using Collins reagent ([CrO_3_](https://en.wikipedia.org/wiki/Chromium_trioxide) with [pyridine](https://en.wikipedia.org/wiki/Pyridine) in CH_2_Cl_2_, 99.1 % yield) ^16,17^. Hydrolysis of **5** was carried out in MeOH and 30% NaOH to yield the ketoacid **6**. The purification of compound **6** was carried out using flash column chromatography. Clemmensen reduction using excess fresh Zn/Hg amalgam in concentrated HCl under reflux conditions yielded compound **7**, 59.0%. Compound **8** (a single enantiomer; purity of 99.1%.) was supplied by Trino Therapeutics Ltd for this study ^9,10,18^. Compounds **9** and **10** were generated from **8** (Scheme S2).

Scheme S1. Synthesis of analogues, 6 & 7 from 3. Reagents and conditions: (i) TMSCH_2_N_2_ (2.7 equiv.) in 2.0 M Et_2_O_,_ RT, 1 h; (ii) [CrO_3_](https://en.wikipedia.org/wiki/Chromium_trioxide) (6.0 equiv.) in dry pyridine (12.0 equiv.) and dry CH_2_Cl_2_ (5 mL), RT, 30 min; (iii) 30% NaOH, 60 ºC, 3 h; (iv) HgCl_2_ (0.1 equiv.), Zn (4.0 equiv.) in H_2_O and concentrated HCl, reflux, 5 h.

Scheme S2. Synthesis of 9 & 10 from 8. Reagents and conditions: (i) [CrO_3_](https://en.wikipedia.org/wiki/Chromium_trioxide) (6.0 equiv.) in dry pyridine (12 equiv.) and dry CH_2_Cl_2_ (5 mL)_,_ RT, 1 h; (ii) HgCl_2_ (0.1 equiv.), Zn (4.0 equiv.) in H_2_O and concentrated HCl, reflux, 8 h.

**4-(((1'*S*,2'*S*)-1'-hydroxy-1',3'-dihydro-1*H*,2'*H*-[2,2'-biinden]-2'-yl)methyl)benzoic acid (PH46 (2))**

Off white solid. ^1^H NMR (400 MHz, CDCl_3_) δH (ppm) 2.87 (1H, *d*, *J* = 13.32 Hz, CH_2_), 3.00 (1H, *d*, *J* = 15.76 Hz, CH_2_), 3.07 (1H, *d*, *J* = 15.72 Hz, CH_2_), 3.29 (1H, *d*, *J* = 13.32 Hz, CH_2_), 3.46 (1H, *d*, *J* = 22.64 Hz, CH_2_), 3.58 (1H, *d*, *J* = 22.60 Hz, CH_2_), 5.27 (1H, *s*, CHOH), 6.49 (1H, *s*, CH=C), 7.00 (2H, *d*, *J* = 7.96 Hz, Ar-H), 7.16-7.34 (6H, *m*, Ar-H), 7.44 (2H, *d,* *J* = 7.08 Hz, Ar-H), 7.90 (2H, *d,* *J* = 7.92 Hz, Ar-H). ^13^C NMR (100 MHz, CDCl_3_) δC (ppm) 38.1 (CH_2_), 38.3 (CH_2_), 39.5 (CH_2_), 55.5 (quat. C), 82.4 (CHOH), 120.2 (tert. C), 123.1 (tert. C), 123.5 (tert. C), 123.9 (tert. C), 124.5 (tert. C), 126.0 (tert. C), 126.6 (tert. C), 126.7 (quat. C), 128.1 (tert. C), 128.2 (tert. C), 2 x 129.2 (2 x tert. C), 2 x 129.9 (2 x tert. C), 140.0 (quat. C), 142.3 (quat. C), 143.2 (quat. C), 144.0 (quat. C), 144.7 (quat. C), 152.0 (quat. C), 171.2 (C=O). HRMS (-H^+^): 381.1490 m/z; required 381.1485; C_26_H_21_O_3_. Achiral HPLC: r.t. 4.8 min, purity 98.1%.

**Methyl (*S*)-4-((1'-oxo-1',3'-dihydro-1*H*,2'*H*-[2,2'-biinden]-2'-yl) methyl)benzoate ((*S*)-5)**

To a vigorously stirred solution of (*R/S*)**-3** (0.10 g, 0.26 mmol, 1.0 equiv.) in a mixture of toluene and MeOH (5 mL, 3:2, *v:v*) was added trimethylsilyldiazomethane (2.0 M in Et_2_O, 0.35 mL, 0.7 mmol, 2.7 equiv.). The addition was in dropwise manner from a syringe until the characteristic yellow colour persisted. TLC analysis was used to confirm complete conversion of (*R/S*)**-3**. On competition, the solvent was removed under reduced pressure yielding (*R/S*)**-4** (0.10 g) as a viscous yellow oil. The crude product was used for the next step reaction without further purification. CrO_3_ (0.15 g, 1.5 mmol, 6.0 equiv.) was added to a solution of dry pyridine (0.24 mL, 0.24 g, 3.0 mmol, 12.0 equiv.) in dry DCM (5 mL). The resulting mixture was stirred at RT for 15 min and a solution of (*R/S*)**-4** (0.10 g, 0.3 mmol, 1.0 equiv.) in dry DCM (0.5 mL) was added rapidly. After 15 min at RT the mixture was decanted, and the remaining tarry solid was extracted with Et_2_O (10 mL). The combined organic extracts were washed with aqueous NaOH (5%, 3 x 10 mL), aqueous HCl (5%, 2 x 10 mL), aqueous NaHCO_3_ (5%, 10 mL) and brine (10 mL). The organic layer was dried over Na_2_SO_4_ and concentrated *in vacuo* yielding (*S*)-**5** (0.10 g, 99.1%) as a pale yellow solid.

Pale yellow solid. ^1^H NMR (400 MHz, CDCl_3_) δH (ppm) 3.32-3.61 (6H, *m*, 3 x CH_2_), 3.89 (3H, *s*, CH_3_), 6.74 (1H, *s*, C=CH), 7.15-7.41 (9H, 2 x *m*, Ar-H), 7.57 (1H, *t*, *J* = 7.69 Hz, Ar-H), 7.75 (1H, *d*, *J* = 8.10 Hz, Ar-H), 7.87 (2H, *d*, *J* = 8.50 Hz, Ar-H). ^13^C NMR (100 MHz, CDCl_3_) δC (ppm) 37.2 (CH_2_), 38.4 (CH_2_), 41.8 (CH_2_), 51.6 (CH_3_), 56.7 (quat. C), 120.3 (tert. C), 123.1 (tert. C), 124.2 (tert. C), 124.3 (tert. C), 125.7 (tert. C), 126.0 (tert. C), 127.3 (tert. C), 128.0 (quat. C), 128.2 (tert. C), 2 x 129.0 (2 x tert. C), 2 x 129.6 (2 x tert. C), 134.7 (tert. C), 2 x 142.5 (quat. C), 142.7 (quat. C), 143.7 (quat. C), 148.3 (quat. C), 151.8 (quat. C), 166.5 (COOCH_3_), 205.2 (C=O). HRMS (+H^+^): 395.1657 m/z; required 395.1642; C_27_H_23_O_3_. Achiral HPLC: r.t. 9.13 min, purity 98.4%.

**(S)-4-((1'-oxo-1',3'-dihydro-1*H*,2'*H*-[2,2'-biinden]-2'-yl)methyl)benzoic acid (*S*-6)**

To a stirred solution of (*S*)-**5** (0.25 g, 0.63 mmol, 1.0 equiv.) in MeOH (2.0 mL) was added aqueous NaOH (30%, 0.67 g, 5.0 mmol, 8.0 equiv.) at RT. The resulting mixture was then heated at 60 °C for 3 h. Water (15 mL) was added and the mixture was washed with dichloromethane (DCM) (2 x 10 mL). The aqueous layer was acidified with aqueous HCl (10%) and extracted with DCM (2 x 10 mL). The combined organic extracts were washed with brine (10 mL) and dried under reduced pressure yielding crude (*S*)-**6** (0.26 g) as an orange viscous oil. Following purification by flash column chromatography using 1:1 ethyl acetate (EtOAc):cyclohexane as eluent (*S*)-**6** was obtained (0.095 g, 96%) as a pale yellow solid.

Pale yellow solid. ^1^H NMR (400 MHz, CDCl_3_) δH (ppm) 3.32-3.62 (6H, *m*, 3 x CH_2_), 6.75 (1H, *s*, CH=C), 7.16 (1H, *t*, *J* = 7.22 Hz, Ar-H), 7.23-7.30 (4H, *m*, Ar-H), 7.36 (1H, *t*, *J* = 7.46 Hz, Ar-H), 7.41 (2H, *d,* *J* = 8.00 Hz, Ar-H), 7.57 (H, *t,* *J* = 7.38 Hz, Ar-H), 7.75 (2H, *d,* *J* = 7.64 Hz, Ar-H), 7.93 (2H, *d,* *J* = 8.04 Hz, Ar-H). ^13^C NMR (100 MHz, CDCl_3_) δC (ppm) 37.2 (CH_2_), 38.4 (CH_2_), 41.8 (CH_2_), 56.7 (quat. C), 120.3 (tert. C), 123.1 (tert. C), 124.2 (tert. C), 124.4 (tert. C), 125.7 (tert. C), 126.0 (tert. C), 127.1 (quat. C), 127.3 (tert. C), 128.3 (tert. C), 2 x 129.6 (2 x tert. C), 2 x 129.7 (2 x tert. C), 134.7 (quat. C), 134.8 (tert. C), 142.6 (quat. C), 143.5 (quat. C), 143.6 (quat. C), 148.2 (quat. C), 151.8 (quat. C), 171.3 (COOH), 205.2 (C=O). HRMS (-H^+^): 379.1329 m/z; required 379.1336; C_26_H_19_O_3_. Achiral HPLC: r.t. 4.29 min, purity 96.1%.

**4-((1',3'-dihydro-1*H*,2'*H*-[2,2'-biinden]-2'-yl)methyl)benzoic acid (7)**

To a stirred mixture of zinc granules (0.58 g, 8.83 mmol, 4.0 equiv.) and HgCl_2_ (59.9 mg, 0.22 mmol, 0.1 equiv.) in water (10 mL) was added conc. HCl (2.0 mL) at RT. The mixture was further stirred for 5 min. Water (10 mL), conc. HCl (10 mL), compound **6** (0.84 g, 2.21 mmol, 1.0 equiv.) and toluene (10 mL) were added subsequently. The resulting mixture was refluxed for 8 h. Conc. HCl (2 mL) was added to the reaction every 2 h, in total 8 mL conc HCl was added over 8 h. The reaction mixture was allowed to cool down to at RT and the layers were separated. The aqueous layer was extracted with DCM (2 x 25 mL). The combined organic extracts were washed with brine (25 mL), dried over Na_2_SO_4_ and concentrated under reduced pressure yielding crude **7** (0.94 g) as a yellow solid. The solids were suspended in EtOAc (5 mL) and filtered. The solids were washed with EtOAc (3 x 2 mL) and dried under reduced pressure yielding **7** methylene acid in moderate yield (0.48 g, 59.0%) as a white solid.

White solid; ^1^H NMR (400 MHz, DMSO-*d*_6_) δ_H_ 3.07 (2H, *s*, CH_2_), 3.08 (2H, *d*, *J* = 15.5 Hz, 2 x C*H*_A_H_B_), 3.15 (2H, *d*, *J* = 15.5 Hz, 2 x CH_A_*H*_B_), 3.46 (2H, *s*, CH_2_), 6.37 (1H, *s*, C=CH), 6.98-7.28 (9H, *m*, Ar-H), 7.39 (1H, d, *J* = 7.0 Hz, Ar-H), 7.73 (2H, *d*, *J* = 8.0 Hz, Ar-H), 12.77 (1H, br *s*, OH); ^13^C NMR (100 MHz, DMSO-*d*_6_) δ_C_ 38.7 (CH_2_), 2 x 43.5 (2 x CH_2_), 44.7 (CH_2_), 51.0 (quat. C), 120.3 (tert. C), 123.3 (tert. C), 123.7 (tert. C), 2 x 124.3 (2 x tert. C), 2 x 126.0 (2 x tert. C), 126.1 (tert. C), 126.9 (tert. C), 128.5 (quat. C), 2 x 128.6 (2 x tert. C), 2 x 129.8 (2 x tert. C), 2 x 141.9 (2 x quat. C), 142.7 (quat. C), 143.8 (quat. C), 144.3 (quat. C), 154.5 (quat. C), 167.1 (COOH). HRMS (-H^+^): 365.1560 m/z; required 365.1547; C_26_H_21_O_2_. Achiral HPLC: r.t. 3.45 min, purity 97.6%.

**(*S*)-2-benzyl-2,3-dihydro-1*H*,1'*H*-[2,2'-biinden]-1-one (9)**

To a stirring solution of **8** (0.140 g, 0.41 mmol) in dry DCM (0.34 mL, 4.2 mmol) and was added a solution of CrO_3_ (0.21 g, 2.10 mmol) in mixture of dry pyridine and dry DCM for 15 min. The reaction was monitored by TLC, which was completed approximately in 1 h. The solution was decanted and extracted with Et_2_O (2 x 10 mL). The fractions were combined, washed with water and brine, dried over NaSO_4_ and evaporated *in vacuo*. The residue was purified by flash column chromatography using 4:1 hexane:ethyl acetate (EtOAc) as eluent to give compound **9** (0.056 g, 40.1%) as a white solid.

White solid; mp: 95−97 ºC. ^1^H NMR (400 MHz, DMSO-*d*_6_) δH (ppm) 3.28-3.35 (2H, *m*, CH_2_), 3.37-3.45 (2H, *m*, CH_2_), 3.46-3.55 (2H, *m*, CH_2_), 6.71 (1H, *s*, CH=C), 7.10-7.28 (8H, *m*, Ar-H), 7.30-7.40 (3H, *m*, Ar-H), 7.49-7.57 (1H, *m*, Ar-H), 7.72 (1H, *d*, *J* = 7.46 Hz, Ar-H). ^13^C NMR (100 MHz, DMSO-*d*_6_) δC (ppm) 37.7 (CH_2_), 38.9 (CH_2_), 42.3 (CH_2_), 57.35 (quat. C), 120.6 (tert. C), 123.5 (tert. C), 124.5 (tert. C), 124.7 (tert. C), 126.1 (tert. C), 126.3 (tert. C), 126.5 (tert. C), 127.5 (tert. C), 2 x 128.2 (2 x tert. C), 2 x 128.4 (2 x tert. C), 130.0 (tert. C), 134.9 (tert. C), 135.3 (quat. C), 137.4 (quat. C), 143.2 (quat. C), 144.3 (quat. C), 149.2 (quat. C), 152.5 (quat. C), 206.0 (C=O). HRMS (+Na^+^): 359.1404 m/z; required 359.1406; C_25_H_20_ONa. Achiral HPLC: r.t. 5.4 min, purity 98.5%.

**2-benzyl-2,3-dihydro-1*H*,1'*H*-2,2'-biindene (10)**

To stirred solution of EtOAc (5 mL) and EtOH (2 mL) was added **9** (0.10 g, 0.30 mmol). The resulting suspension was heated to 70 ºC and a 30% solution of NaOH was added slowly drop-wise (2 mL) over approximately 20 min. After reflux for 4 h, the mixture was cooled and saturated NH_4_Cl was added to quench the reaction. The solution was extracted with EtOAc (3 x 15 mL) and the organic fractions were collected. The aqueous layer was acidified using 2 M HCl. The acidified aqueous layer was further extracted with EtOAc (3 x 30 mL). The fractions were combined, washed with water and brine, dried over NaSO_4_ and evaporated *in vacuo*. The residue was purified by flash column chromatography on silica gel 230-400 mesh using 3:1 (hexane:EtOAc) as eluent to give target compound **10** (89.5 mg, 92.6%) as a pale white solid.

Pale white solid; mp: 87−89 ºC. ^1^H NMR (400 MHz, DMSO-*d*_6_) δH (ppm) 3.00 (2H, *s*, CH_2_), 3.16 (4H, *m*, *J* = 5.81 Hz, 2 x CH_2_), 3.46 (2H, *s*, CH_2_), 6.43 (1H, *s*, CH=C), 6.88-6.94 (2H, *m*, Ar-H), 7.18-7.29 (11H, *m*, Ar-H). ^13^C NMR (100 MHz, DMSO-*d*_6_) δC (ppm) 29.7 (CH_2_), 39.4 (CH_2_), 43.9 (CH_2_), 45.4 (CH_2_), 51.1 (quat. C), 120.4 (tert. C), 123.4 (tert. C), 123.9 (tert. C), 2 x 124.6 (2 x tert. C), 126.1 (tert. C), 126.3 (tert. C), 126.4 (tert. C), 2 x 127.3 (2 x tert. C), 2 x 127.7 (2 x tert. C), 128.5 (tert. C), 128.6 (tert. C), 130.1 (quat. C), 138.5 (quat. C), 142.3 (quat. C), 142.9 (quat. C), 145.0 (quat. C), 154.9 (quat. C). HRMS (-Cl^+^): 321.1652 m/z; required 321.1649; C_25_H_21_. Achiral HPLC: r.t. 6.4 min, purity 87.8%.

**(1*S*,2*S*)-2-benzyl-2,3-dihydro-1*H*,1'*H*-[2,2'-biinden]-1-ol (8)**

Pale yellow solid. ^1^H NMR (400 MHz, CDCl_3_) δH (ppm) 2.78 (1H, *d,* *J* = 13.36Hz, CH_2_), 2.99 (1H, *d,* *J* = 15.40Hz, CH_2_), 3.10 (1H, *d,* *J* = 15.60Hz, CH_2_), 3.22 (1H, *d,* *J* = 13.44Hz, CH_2_), 3.46 (1H, *d,* *J* = 22.52Hz, CH_2_), 3.59 (1H, *d,* *J* = 22.48Hz, CH_2_), 5.25 (1H, *s*, CHOH), 6.51 (1H, *s*, CH=C), 6.89-6.91 (2H, *m*, Ar-H), 7.17-7.18 (4H, *m,* Ar-H), 7.27-7.32 (5H, *m,* Ar-H), 7.44 (2H, *d,* J=7.36Hz, Ar-H). ^13^C NMR (100 MHz, CDCl_3_) δC (ppm) 2 x 38.0 (2 x CH_2_), 39.6 (CH_2_), 55.4 (quat. C), 82.7 (CHOH), 120.0 (tert. C), 123.1 (tert. C), 123.5 (tert. C), 123.6 (tert. C), 124.5 (tert. C), 125.7 (tert. C), 125.9 (tert. C), 126.5 (tert. C), 2 x 127.4 (2 x tert. C), 127.8 (tert. C), 128.0 (tert. C), 2 x 129.7 (2 x tert. C), 137.9 (quat. C), 140.3 (quat. C), 142.5 (quat. C), 143.4 (quat. C), 144.3 (quat. C), 152.7 (quat. C). HRMS (+Na^+^): 361.1568 m/z; required 361.1563; C_25_H_22_ONa. Achiral HPLC: r.t. 4.5 min, purity 99.1%.
